# Resection in the popliteal fossa for metastatic melanoma

**DOI:** 10.1186/1477-7819-5-8

**Published:** 2007-01-19

**Authors:** Ugo Marone, Corrado Caracò, Maria Grazia Chiofalo, Gerardo Botti, Nicola Mozzillo

**Affiliations:** 1 Department of Surgical Oncology "B", National Cancer Institute of Naples, Italy; 2 Department of Pathology, National Cancer Institute of Naples, Italy

## Abstract

**Background:**

Traditionally metastatic melanoma of the distal leg and the foot metastasize to the lymph nodes of the groin. Sometimes the first site of nodal disease can be the popliteal fossa. This is an infrequent event, with rare reports in literature and when it occurs, radical popliteal node dissection must be performed.

**Case presentation:**

We report a case of a 36-year old man presented with diagnosis of 2 mm thick, Clark's level II-III, non ulcerated melanoma of the left heel, which developed during the course of the disease popliteal node metastases, after a superficial and deep groin dissection for inguinal node involvement. Five months after popliteal lymph node dissection he developed systemic disease, therefore he received nine cycles of dacarbazine plus fotemustine. To date (56 months after prior surgery and 11 months after chemotherapy) he is alive with no evidence of disease.

**Conclusion:**

In case of groin metastases from melanoma of distal lower extremities, clinical and ultrasound examination of ipsilateral popliteal fossa is essential. When metastatic disease is found, radical popliteal dissection is the standard of care. Therefore knowledge of anatomy and surgical technique about popliteal lymphadenectomy are required to make preservation of structures that if injured, can produce a permanent, considerable disability.

## Background

Involvement of the regional lymph nodes is the most important predictor of outcome in patients with cutaneous melanoma [1]. Traditionally metastatic melanoma of the distal leg and the foot metastasize to the lymph nodes of the groin. Sometimes the first site of nodal disease can be the popliteal fossa. This is an infrequent event, with rare reports in literature and when it occurs, radical popliteal node dissection must be performed [2]. We report a case of patient with melanoma on the heel, which developed popliteal node metastases during the course of the disease, after a superficial and deep groin dissection for inguinal node involvement.

## Case presentation

**Figure 1:**
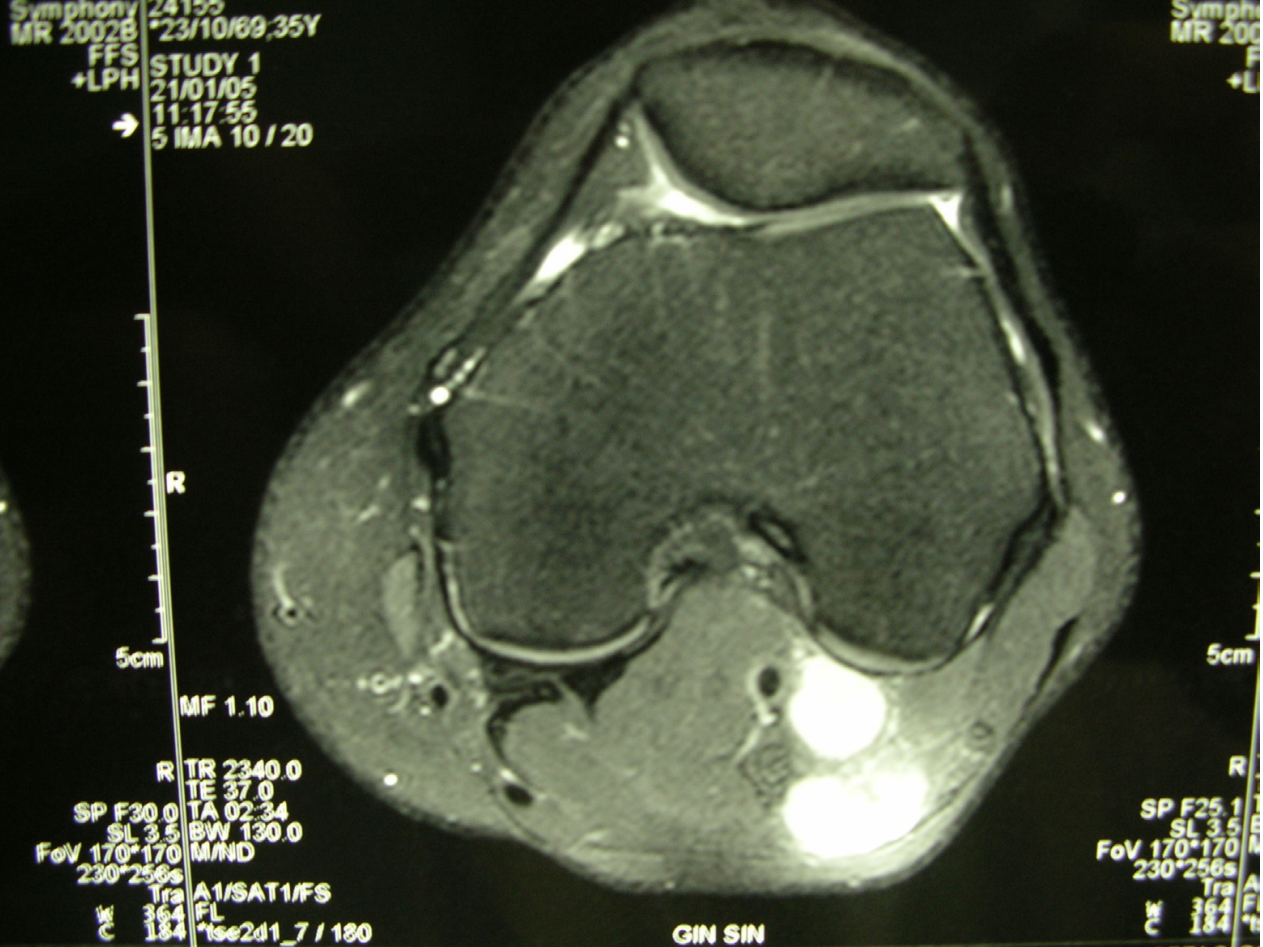
Axial MRI of the left popliteal fossa showing two enlarged nodes in close proximity to the popliteal vessels.

**Figure 2:**
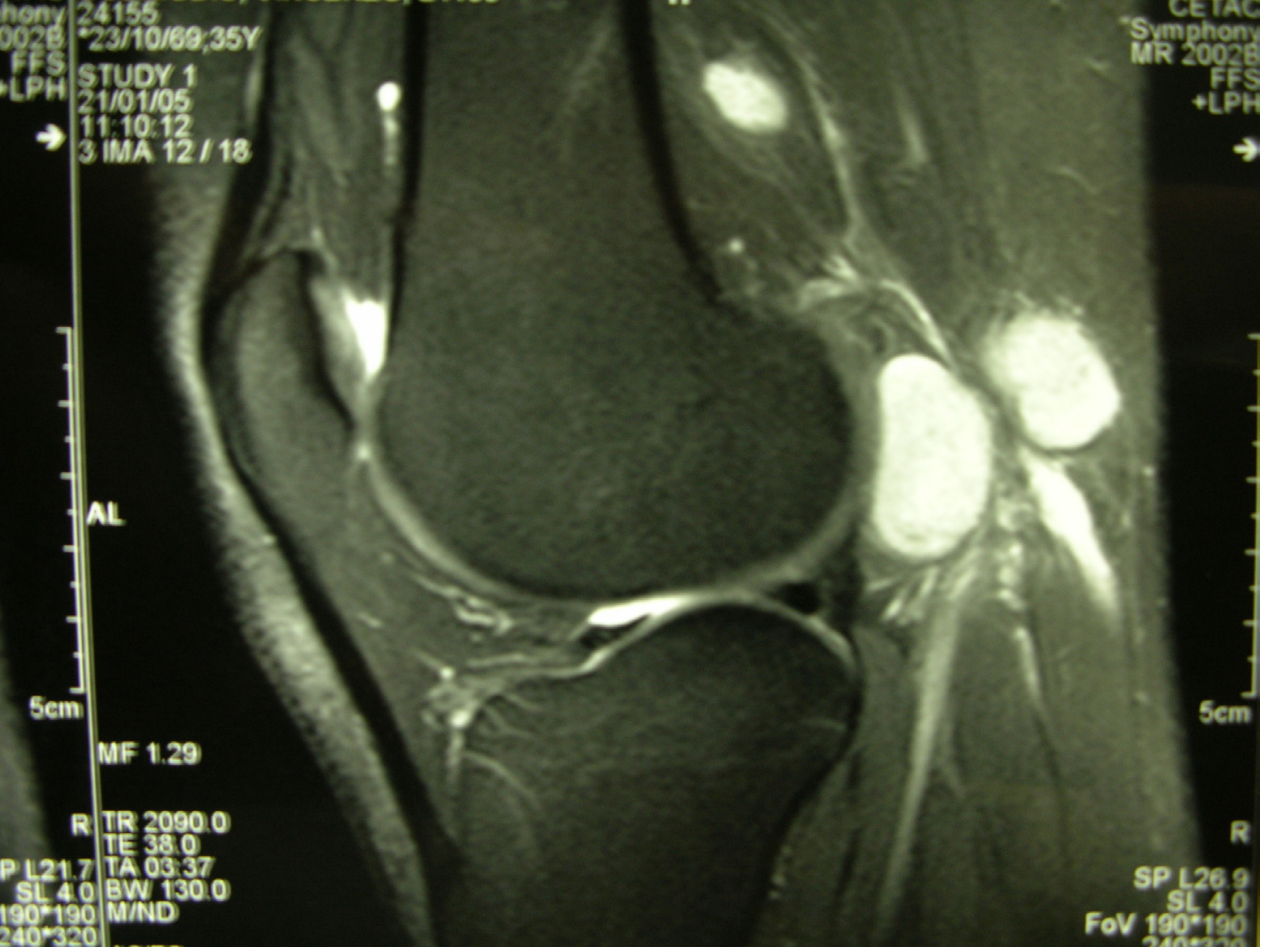
Sagittal MRI of the left popliteal fossa showing two involved nodes anterior to the neurovascular bundle.

**Figure 3:**
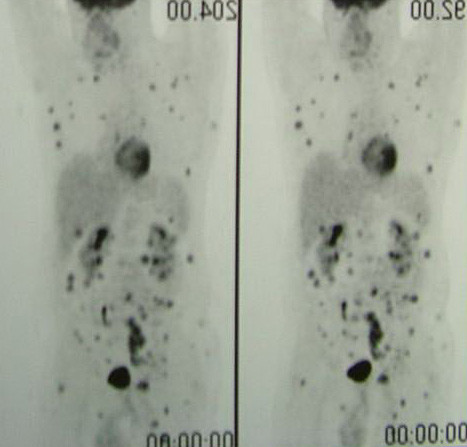
PET whole-body showing systemic uptake of the radiotracer.

**Figure 4:**
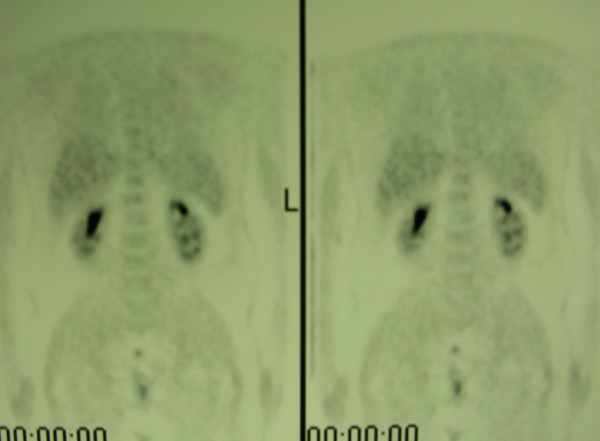
PET whole-body performed after chemotherapy, showing no signs of systemic disease.

**Figure 5:**
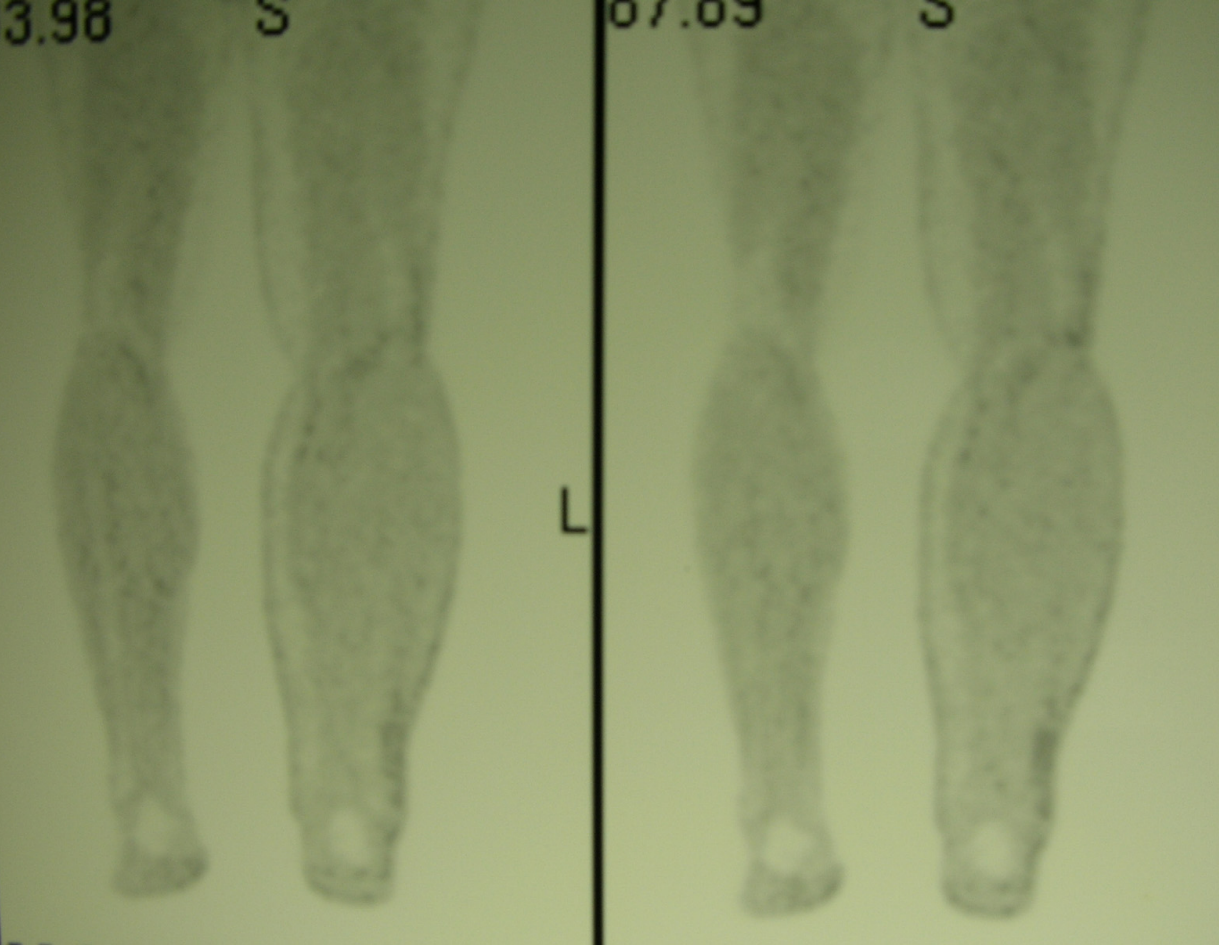
PET whole-body performed after chemotherapy, showing no evidence of disease in the left popliteal fossa.

In January 2002, a 36-year old man presented with diagnosis of 2 mm thick, Clark's level II-III, non ulcerated melanoma of the left heel. He underwent wide local excision and sentinel lymph node biopsy (SLNB). Preoperative lymphoscintigraphy showed uptake of the radiotracer in three nodes of ipsilateral groin that after removal resulted on final pathology negative for metastatic melanoma. In the February 2005 the patient presented at follow-up with clinical nodal disease in the left groin confirmed by fine needle aspiration cytology (FNAC). Preoperative staging did not reveal signs of systemic disease, therefore the patient underwent an inguinal lymph node dissection, that revealed 1 positive lymph node out of 8, and concomitant iliac and obturator lymph node dissection, with 3 positive nodes out of 11. Three months later he developed a mild-grade lymphedema of the inferior left extremity. Ultrasounds study (US) and magnetic resonance imaging (MRI) (Figure 1, 2) revealed evidence of two involved nodes in the popliteal fossa measuring respectively 2.7 × 1.7 and 2.3 × 1.5 cm, positive for metastatic melanoma by FNAC. Therefore, the patient underwent popliteal lymph node dissection according to the technique first described by Karakousis [3]. In the October 2005 during follow-up control, whole body positron emission tomography (PET) showed uptake of the radiotracer in the superficial tissues of the trunk, left thigh, left leg and in the abdomino-pelvic area (Figure 3). Computerised tomography (CT) was negative for metastatic disease. The patient so underwent explorative laparotomy that revealed peritoneal carcinosis confirmed by pathology. After surgery he received chemotherapy (fotemustine 100 mg/m^2 ^plus dacarbazine 900 mg/m^2 ^intravenously, every three weeks, for a total of nine cycles) and in September 2006 the patient is alive with no evidence of disease (Figure 4, 5).

## Discussion

The evidence of positive nodes in the popliteal fossa in patients with metastatic melanoma of the lower leg and the foot is a rare event with a paucity of reports in the literature, due to the difficulty in examining the popliteal fossa, where the lymph nodes lie deep to the fascia and are not easily palpable [2]. For this both patient and surgeon might miss the popliteal metastasis until the patient dies of distant metastasis [4]. The use of lymphoscintigraphy alone or in combined with blue dye and a handheld gamma probe makes possible to delineate the percentage of drainage into popliteal nodes ranging from 1% to 20% in patients with malignant melanoma of the lower extremities [5]. Despite the wide variability of sentinel node detected in the popliteal fossa by lymphoscintigraphy, popliteal metastasis are extremely rare. From 1996 to 2005 at the National Cancer Institute of Naples, 980 patients with diagnosis of cutaneous melanoma were submitted to SLNB. Of these, 148 had primary lesion in the lower extremities (15.1%), 108 at level of the distal leg (72.9%) and 40 at level of the foot (27%). In all the patients lymphoscintigraphy showed drainage to the inguinal nodes except in two cases (1.3%) in which first site of drainage was the popliteal fossa, both negative for metastatic melanoma at pathology. Only in the case reported we recognized clinical metastatic disease in the popliteal area (0.7%).

Thompson *et al*., reported a retrospective study on 4262 patients with primary melanoma of the distal lower limb from the Sydney cancer database. Thirteen of 4262 patients (0.3%) were recorded as having developed metastatic disease in a popliteal node. Five of 13 patients with popliteal disease had metastatic inguinal node at the time or before the presentation of popliteal disease. Eight of the 13 patients with popliteal node metastases subsequently developed distant metastatic disease, at mean interval of 39 months (range 3–127 months) from the time of treatment of their primary melanomas. At last follow-up reported (median 97 months, range 2–339 months) seven of the patients had died of melanoma and six were alive, all clinically disease free. Thompson demonstrated that if a node in the popliteal fossa is observed on lymphoscintigraphy, a sentinel lymph node biopsy is indicated. If the specimen is positive for melanoma, a popliteal node dissection is the logical next step. So they concluded that there are two indications for popliteal node clearance: a histologically positive sentinel node in the popliteal fossa or clinical evidence of metastatic disease in this area [6]. Menes *et al*., recognized 106 patients with melanoma at the knee or distally. Ten patients (9%) had drainage to the popliteal basin at lymphoscintigraphy. They identified a 2.8% rate of metastatically involved popliteal nodes. Metastasis was detected in one patient by immunohistochemical analysis and were palpable in two at presentation. All had concomitant drainage to the groin, evident clinically or by lymphoscintigraphy. In the patient, with positive popliteal sentinel lymph node, groin sentinel node was disease free, so he underwent radical popliteal dissection. Two patients, with synchronous palpable popliteal and groin metastases, underwent radical popliteal and groin dissection. All 3 patients relapsed a short time after surgery with systemic and in-transit metastases; all 3 subsequently died. The authors concluded that popliteal area should always be examined when the primary lesion involves the distal lower extremities and when metastatic disease is found, radical popliteal dissection is the standard of care [5].

In the extremities drainage of cutaneous melanoma is considered more predictable than in the head and neck [7]. Usually two ways of drainage are described for the distal lower extremities: the principal which originates at the medial aspect of the foot, draining into the inguinal nodes; the secondary which originates at the lateral aspect of the foot, draining into popliteal nodes [8].

Previous authors have suggested that sites which drain to the popliteal nodal basin are located on the posterolateral aspect of the heel, below the lateral malleolus. However, Thompson *et al*., showed that positive nodes in the popliteal fossa can be expected from a lesion located anywhere below the knee [6].

In our case primary site was at the postero lateral aspect of the left heel. At presentation there was no evidence of clinical disease in the ipsilateral groin or popliteal fossa. SLNB showed three negative nodes. Recurrence after tumour-negative SLNB may result from an error in the technique of sentinel node detection at the time of lymphoscintigraphy or the operation, an inaccurate histopathological stain, or the biologic features of the melanoma [9]. In our case popliteal nodes could correspond to the first site of recurrence, while the inguinal nodes the subsequent, although the clinical evidence of groin involvement seems to demonstrate the contrary, so the negative sentinel node was due to a missed identification of popliteal drainage at the time of the lymphoscintigraphy. Studies on lymphoscintigrams show that whenever there is lymphatic drainage to the popliteal fossa, it is accompanied by drainage to the groin, which can be primary or secondary drainage site. However, the popliteal nodes may be metastatic in the absence of a detectable groin metastasis [5]. Hatta *et al*., considered that in case of patients with popliteal involvement having metastatic inguinal nodes at the time or before the presentation of popliteal disease, popliteal metastasis could represent "backflow" from inguinal metastasis or in transit metastasis [4].

The utilitarian surgical approach to the popliteal fossa involves a Z-plasty incision, to obtain optimal exposure of diamond shaped popliteal space. The initial step of popliteal dissection is adequate exposure of the popliteal fossa and identification of the neurovascular bundle. Identification of the popliteal fascia, which is very thin and friable, is a basic surgical step. This structure serves as a landmark because of its close proximity to the neurovascular bundle. As the fascia is exposed, the most superficial key anatomic structures are the lesser saphenous vein, that must be ligated and divided, and some small cutaneous nerve terminal branches. Next the incision of the deep fascia must be careful because of the superficiality of the nerves. The tibial nerve is the most superficial structure within the popliteal fossa (the popliteal vessels lie below the nerve) likewise the common peroneal nerve. A fat pad overlying and alongside the popliteal vessels is reported to enclose two to seven lymph nodes [10]. With careful dissection, the fat pad and lymphatic tissue are removed in continuity superficial to, below, and along the neurovascular bundle. In our case, the presence of two voluminous lymph nodes created distortion of the anatomic planes with compression of the neurovascular bundle, making particularly difficult the identification of the key anatomic structures and the dissection. After adequate haemostasis and reapproximation of the deep fascia, the wound is closed over closed-suction drain. To reduce tension on the suture lines, and avoids significant oedema, the lower extremity is kept elevated in 15–20° of flexion in a posterior splint or a knee immobilizer for few days before return to usual ambulation.

## Conclusion

The case described was peculiar in the time of appearance of popliteal and groin metastases. Although the clinical evidence of groin involvement come as first site of recurrence, it must be considered as a consequence of popliteal metastases probably present since the sentinel node biopsy. In all cases of groin metastases from melanoma of distal lower extremities, clinical and ultrasound examination of ipsilateral popliteal fossa is essential, especially where the foot is the most frequent site of malignant melanoma, like in Japan [11]. When metastatic disease is found, radical popliteal dissection is the standard of care. Therefore knowledge of anatomy and surgical technique about popliteal lymphadenectomy is indispensable to make preservation of structures that if injured, can produce a permanent, considerable disability.

## Competing interests

The author(s) declare that they have no competing interests.

## Authors' contributions

**UM **conceived the study, carried out the literature search, and draft the manuscript; **CC **helped in management of the patient and preparation of the manuscript; **MGC **and **GB **carried out literature review and manuscript drafting; **NM **shaped the idea for the manuscript, coordinated the study and edited the manuscript.

All authors read and approved the final manuscript.
